# Prognostic Value of Dynamic Lactate Dehydrogenase Trends in Immunotherapy for Advanced Esophageal Squamous Cell Carcinoma: Retrospective Cohort Study

**DOI:** 10.2196/73576

**Published:** 2026-01-14

**Authors:** Baidong Zhang, Zhichao Kang, Yi Ding, Wang Jing, Alei Feng, Renya Zeng, Jianan Li, Yi Zhao, Yuanliu Nie, Wentao Zhang, Lu Sun, Zhe Yang

**Affiliations:** 1 Tumor Research and Therapy Center, Shandong Provincial Hospital Affiliated to Shandong First Medical University, 324 Jingwu Road, Jinan, 250000, China, 86 0531-87938911; 2 Department of Radiation Oncology, Shandong Provincial Hospital, Jinan, China

**Keywords:** lactate dehydrogenase, esophageal squamous cell carcinoma, immune checkpoint inhibitors, prognostic value, biomarker

## Abstract

**Background:**

Immune checkpoint inhibitors (ICIs) have emerged as a pivotal treatment for advanced esophageal squamous cell carcinoma (ESCC). However, their efficacy can significantly differ among patients, highlighting the need for reliable prognostic markers to enhance treatment outcomes. Lactate dehydrogenase (LDH) plays a key regulatory role in the complex relationship between cancer metabolism and the immune system, suggesting that monitoring LDH levels may provide valuable insights into treatment efficacy and inform personalized therapeutic strategies for advanced ESCC.

**Objective:**

This study aimed to explore the prognostic significance of dynamic changes in LDH levels during ICI therapy in predicting treatment outcomes.

**Methods:**

We retrospectively analyzed the clinical data of 126 patients with advanced ESCC who received first-line ICI therapy at the Department of Radiation Oncology, Cancer Center, Shandong Provincial Hospital, between April 2018 and November 2022. Serum LDH levels were measured after every 3 cycles of combined immunotherapy and chemotherapy. Receiver operating characteristic curve analysis determined the optimal LDH reduction threshold. Kaplan-Meier survival curves and Cox regression models assessed progression-free survival (PFS) and overall survival.

**Results:**

Among the 126 patients, 55 (43.6%) were classified into the LDH-increased group, while 71 (56.4%) belonged to the LDH-decreased group. Within the LDH-increased group, 78.2% (43/55) of the patients were male, compared to 90.1% (64/71) in the LDH-decreased group. The median age of patients in the LDH-increased group was 59 (range 55‐68) years, whereas the median age in the LDH-decreased group was 65 (range 58‐65) years. LDH decrease following first-line ICI therapy was associated with improved outcomes compared to LDH increases (median PFS 13.4, IQR 8.1‐24.3 mo vs median 10.8, IQR 4.8‐20.6 mo; *P*= .03). Patients with a posttreatment LDH decrease of more than 14.4% had a median PFS of 11.1 (IQR 7.2‐24.3) months, whereas those with an LDH decrease between 0% and 14.4% had a median PFS of 21.7 (IQR 9.4‐34.5) months. Conversely, an increase in LDH resulted in a median PFS of 10.8 (IQR 4.8‐20.6) months. Patients with tumor reduction exhibited a significantly greater decrease in LDH levels compared with those without tumor reduction (*P*<.001). Multivariate analysis identified LDH decrease as an independent predictor of a 41% lower mortality risk (hazard ratio 0.59, 95% CI 0.36‐0.96; *P*=.04).

**Conclusions:**

In patients with advanced ESCC, a decrease in serum LDH levels ranging from 0% to 14.4% after treatment initiation was significantly associated with prolonged PFS. Notably, an early decrease in LDH levels observed after 3 cycles of immunotherapy further correlated with improved clinical outcomes. These results highlight the potential of LDH as a valuable biomarker for risk stratification and personalized treatment optimization in advanced ESCC.

## Introduction

### Background

Esophageal squamous cell carcinoma (ESCC) is a globally prevalent oncological challenge marked by high incidence and mortality rates, particularly in regions such as Asia, Eastern Europe, and Africa [1]. The typically asymptomatic nature of early-stage ESCC often results in late-stage diagnoses, which limit therapeutic options and contribute to high mortality rates [2]. Current management strategies for ESCC include a combination of surgery, radiation, and chemotherapy. However, the 5-year survival rate remains dismally low at approximately 20%, emphasizing the aggressive nature of this malignancy and the complexities surrounding its treatment [3].

Recent advancements in immunotherapy, particularly immune checkpoint inhibitors (ICIs) targeting the programmed death-1 (PD-1) and programmed death-ligand 1 (PD-L1) axis, such as nivolumab and pembrolizumab, have revolutionized the treatment landscape for advanced ESCC. Evidence from pivotal studies such as KEYNOTE-181 and ATTRACTION-3 has demonstrated the superior efficacy of ICIs compared to conventional chemotherapy [4]. Despite these breakthroughs, not all patients with advanced ESCC derive significant benefits from ICIs [5], highlighting the critical need for reliable, accessible, and cost-effective biomarkers to predict treatment response and patient prognosis more effectively.

Lactate dehydrogenase (LDH), a key enzyme in cancer cell metabolism, has gained attention as a potential prognostic biomarker in oncology due to its role in metabolic reprogramming and tumor adaptation to hypoxic microenvironments [6]. Elevated LDH levels have been linked to increased tumor aggressiveness, metabolic stress, and poor clinical outcomes across various malignancies, including melanoma, lung cancer, and breast cancer, underscoring its potential as a universal biomarker of malignancy [7,8]. Emerging research highlights the intricate relationship between cancer metabolism and the immune system, with LDH functioning as a critical mediator of this interplay [9]. LDH plays a pivotal role in metabolic reprogramming, enabling tumor survival in hypoxic conditions by facilitating anaerobic glycolysis and lactate production. These processes not only support tumor growth but also contribute to the creation of an immunosuppressive tumor microenvironment. This suggests that dynamic changes in LDH levels could significantly influence the efficacy of immunotherapeutic approaches in ESCC [10]. Preliminary studies have shown that variations in LDH levels following ICI treatment correlate with improved survival outcomes, pointing to the potential of LDH as a valuable prognostic tool in ESCC immunotherapy [11].

### Objectives

To identify a more accessible and precise prognostic marker for advanced ESCC, this study enrolled patients receiving first-line immunotherapy combined with chemotherapy. The primary objective was to investigate the prognostic value of dynamic changes in LDH by assessing LDH kinetics. This research aims to provide new insights into the potential of LDH as a cost-effective and practical biomarker for guiding personalized treatment strategies and improving outcomes in patients with advanced ESCC.

## Methods

### Ethical Considerations

This study was approved by the Ethics Committee of Biomedical Research at Shandong Provincial Hospital (SWYX NO 2023‐595). Given the retrospective nature of the study, which involved secondary analysis of existing electronic health records, no prospective informed consent specific to this research was required. At the time of admission or outpatient registration, all patients provided a general consent permitting the use of their medical records for research purposes, in accordance with institutional policy. All data in this study were deidentified to ensure participant anonymity and protect confidentiality. This was a retrospective study based on the analysis of pre-existing patient medical records. As such, no participants were actively recruited for this research, and therefore, no compensation was involved.

### Patient Selection

This retrospective cohort study was conducted at Shandong Provincial Hospital between April 2018 and November 2022. The study included patients diagnosed with stage IV ESCC based on the Tumor-Node-Metastasis classification system. Eligible patients were those who received ICIs in combination with chemotherapy and who had a Karnofsky Performance Status score greater than 80.

The exclusion criteria were as follows: history of other malignancies, presence of autoimmune disorders, previous immunotherapy with agents such as Carrelizumab or Tislelizumab, and incomplete clinical or laboratory data.

The follow-up period ended in September 2023. The medical records were retrieved from the Shandong Provincial Hospital database. Data collected included patient age, sex, comorbidities, tumor location, treatment response evaluations, TNM stages, stage at initial diagnosis, baseline LDH levels, posttreatment LDH levels, and dynamic changes in LDH levels.

### Treatment and Evaluation Criteria

All participants in the study received a treatment regimen consisting of PD-1 inhibitors combined with chemotherapy agents, such as 5-fluorouracil, cisplatin, taxanes, and irinotecan, administered either as monotherapy or as part of polychemotherapy protocols. The PD-1 inhibitors were administered intravenously at a standard dose of 200 mg, with infusions performed every 2 to 3 weeks for a total of 4 to 6 cycles, or until disease progression, unacceptable toxicity, or death.

Serum LDH levels were measured at 2 key time points: at baseline (before the initiation of immunotherapy) and after the final immunotherapy cycle. LDH quantification was performed by the Department of Laboratory Medicine at Shandong Provincial Hospital. The study evaluated treatment efficacy longitudinally, with key end points including progression-free survival (PFS) and overall survival (OS). Progressive disease was defined as a ≥20% increase in the sum of the longest diameters of target lesions from the nadir, the appearance of new measurable disease foci, or other substantial indicators of disease progression, according to the Response Evaluation Criteria in Solid Tumors (version 1.1) [12]. PFS was measured from the initiation of anti–PD-1 therapy to the point of documented disease progression or death from any cause, while OS was defined as the time from the initiation of immunotherapy to death from any cause.

### Statistical Analysis

#### Overview

Descriptive statistics were used to summarize baseline characteristics, using means (SD) for normally distributed variables, medians (IQR) for non–normally distributed variables (as assessed by the Shapiro-Wilk test), and frequencies (%) for categorical variables. Serum LDH levels were measured both before treatment and after immunotherapy (median of 3 cycles). The LDH reduction rate was calculated as follows:


$$\left[\frac{\text{Baseline LDH}-\text{Post-treatment LDH}}{\text{Baseline LDH}}\right]\times 100\%$$

#### Critical Methodological Note on Cutoff Selection

The initial receiver operating characteristic analysis for OS prediction yielded suboptimal performance (area under the curve=0.602; data not shown), likely due to the heterogeneity inherent in retrospective cohorts. Consequently, we adopted a distribution-driven approach, selecting the 25th percentile of observed LDH reduction values (−14.4%) as the primary cutoff. This threshold, representing a substantial decline in LDH, demonstrated significant discriminative power for PFS in subsequent analyses.

Kaplan-Meier curves, analyzed using log-rank tests, were used to compare survival between different LDH trajectory groups. Cox regression analysis was used to identify prognostic factors, with a two-step process: (1) univariate screening with a *P*<.10 entry threshold and (2) multivariate adjustment for age, Karnofsky Performance Status, and TNM stage. Results were reported as adjusted hazard ratios (HRs) with 95% CIs. Missing data were minimal (<1% for LDH, PFS, and OS) and were handled using complete-case analysis, confirmed to be missing completely at random; sensitivity analyses with multiple imputation yielded consistent results. The sample size (n=126) provided 80% power (*α*=.05) to detect an HR of 0.60 for PFS improvement in LDH reducers versus nonreducers, based on KEYNOTE-181 data with a 15% attrition adjustment. Analyses were conducted using SPSS (version 25.0; IBM Corp), with statistical significance set at *P*<.05 (2-tailed).

## Results

### Patient Characteristics

**Figure 1.**
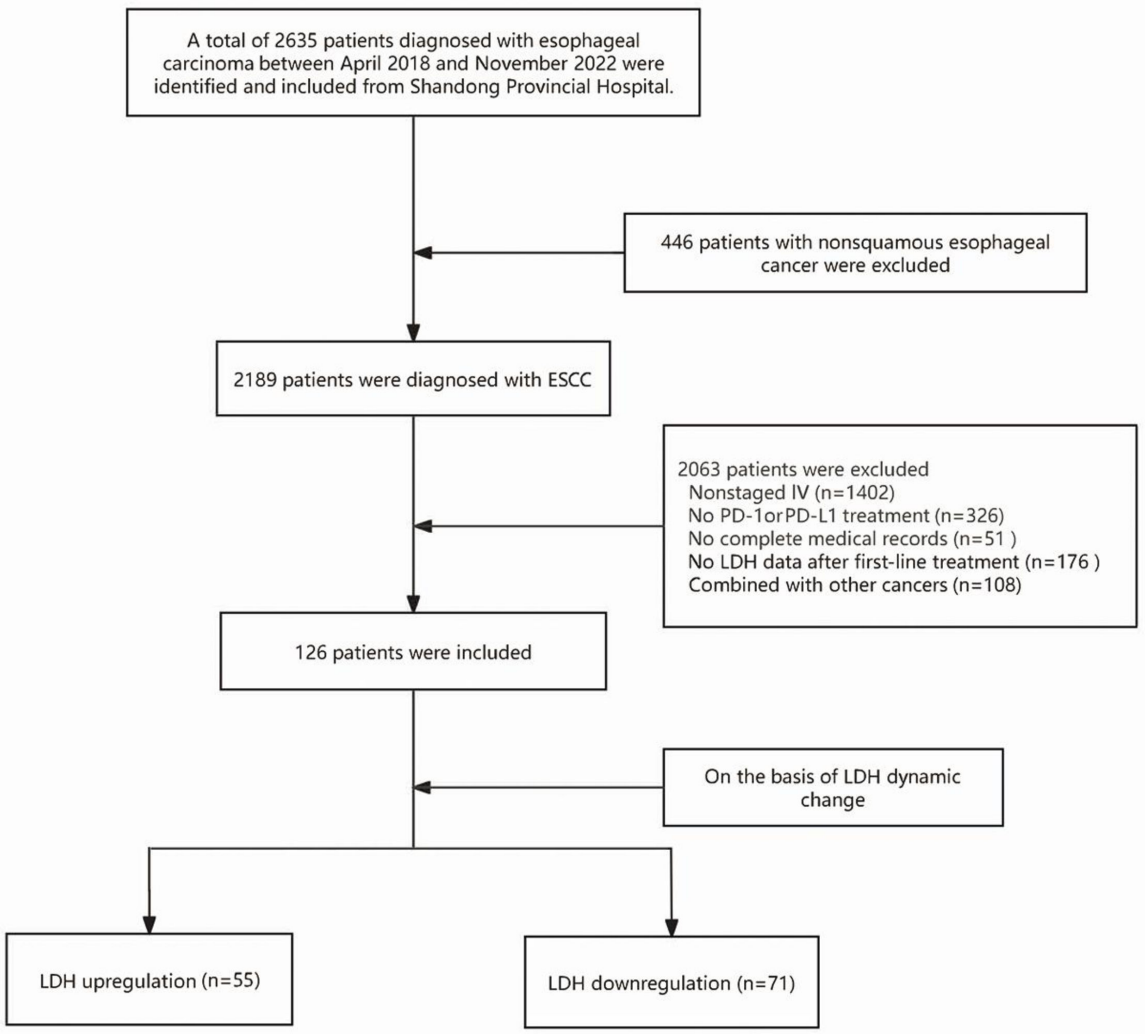
Patient selection criteria and process. ESCC: esophageal squamous cell carcinoma; LDH: lactate dehydrogenase; PD-1: programmed death-1; PD-L1: programmed death-ligand 1.

As shown in Figure 1, from an initial cohort of 2635 patients diagnosed with ESCC, 446 (16.9%) patients with non-ESCC diagnoses, 1402 (53.2%) patients at non-IV stages of ESCC, 326 (12.4%) patients who did not receive PD-L1 and PD-1 treatment, 51 (1.94%) patients with incomplete medical records, 176 (6.68%) patients lacking LDH data during first-line treatment, and 108 (4.1%) patients diagnosed with other cancers were excluded. The final study cohort consisted of 126 patients.

The follow-up period concluded with a median follow-up duration of 13.8 (range 8.00‐18.75) months. From the 126 patients, 9 (7.14%) were alive at the time of follow-up. The median PFS for all patients was 11.8 (95% CI 6.1‐22.9) months, while the median OS was 28 (95% CI 14.6‐43.9) months.

Patient characteristics are summarized in Table 1. Among the 126 patients, 55 (43.6%) were classified into the LDH-increased group, while 71 (56.4%) belonged to the LDH-decreased group. Within the LDH-increased group, 78.2% (43/55) of the patients were male, compared to 90.1% (64/71) in the LDH-decreased group. The median age of patients in the LDH-increased group was 59 (range 55‐68) years, whereas the median age in the LDH-decreased group was 65 (range 58‐65) years. In addition, in the LDH-increased group, 38.2% (21/55) of patients had other comorbidities, whereas in the LDH-decreased group, the proportion was 43.7% (31/71).

The LDH ratio was calculated by comparing the posttreatment LDH levels to the baseline LDH levels. On the basis of this ratio, patients were subsequently classified into 2 categories: those with elevated LDH levels and those with decreased LDH levels.

**Table 1.** Patient characteristics—comparison of lactate dehydrogenase (LDH) levels after treatment and at baseline.

	LDH upregulation (n=55), n (%)	LDH downregulation (n=71), n (%)	*P* value
Age (years), median (IQR)	59 (55‐68)	65 (58‐65)	—^a^
Age (years), n (%)			.07
≤63	33 (60.0)	30 (42.3)	
>63	22 (40.0)	41 (57.7)	
Gender, n (%)			.08
Male	43 (78.2)	64 (90.1)	
Female	12 (21.8)	7 (9.9)	
Tumor location, n (%)			.81
Upper	5 (9.1)	5 (7.0)	
Middle	28 (50.9)	40 (56.3)	
Lower	22 (40.0)	26 (36.6)	
T stage, n (%)			.59
T1-3	25 (45.4)	28 (39.4)	
T4	30 (54.6)	43 (60.6)	
N stage, n (%)			.24
N0-1	8 (14.5)	5 (7.0)	
N2-3	47 (85.5)	66 (93.0)	
Clinical stage, n (%)			.57
IVA	16 (29.1)	25 (35.2)	
IVB	39 (70.9)	46 (64.8)	
Comorbidity, n (%)			.07
Yes	21 (38.2)	31 (43.7)	
No	34 (61.8)	40 (56.3)	

a Not applicable.

### Kaplan-Meier Survival Analysis

**Figure 2.**
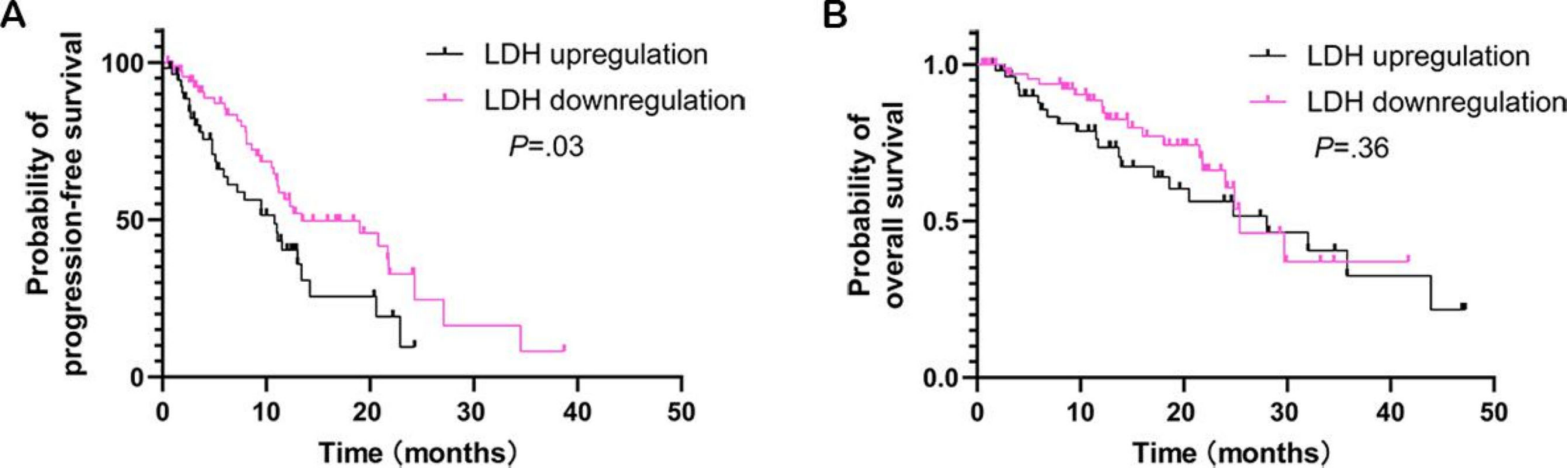
Kaplan-Meier curves for (A) progression-free survival and (B) overall survival based on lactate dehydrogenase (LDH) dichotomization (downregulation vs upregulation). Patients with downregulated LDH levels exhibited a significantly better prognosis.

To assess dynamic changes in LDH levels, collection points were established at intervals of every 3cycles of chemotherapy combined with immunotherapy. Dichotomous analysis revealed that patients exhibiting downregulated LDH levels showed a significant improvement in PFS (*P*=.03; Figure 2A). However, no statistically significant difference was observed in OS (*P*=.36; Figure 2B).

**Figure 3.**
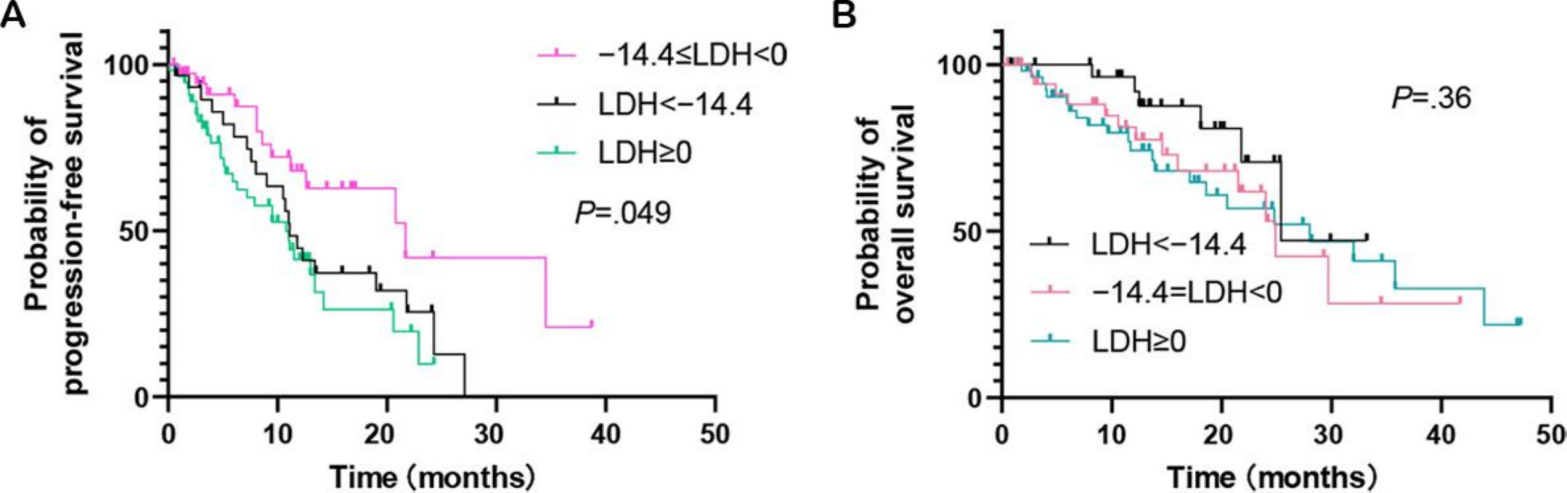
Kaplan-Meier curves of (A) progression-free survival and (B) overall survival stratified by lactate dehydrogenase (LDH) change into 3 groups: an increase (LDH≥0%), a moderate decrease (−14.4%≤LDH<0%), and a marked decrease (LDH<−14.4%) following treatment. A decrease in LDH levels exceeding 14.4% after treatment was associated with a significantly improved prognosis.

It was noted that a reduction in LDH levels may also reflect a decline in the patient’s physical condition or nutritional status, necessitating further stratified analysis. Receiver operating characteristic curve methodology was applied, with OS used as the end point criterion for analysis. On the basis of this methodology, an appropriate threshold for LDH reduction was identified as greater than −14%, closely approximating the −14.4% mark, which represents the boundary at the 25th percentile of the data distribution. At the threshold of −14.4%, a significant difference in PFS was observed (Figure 3A), while no significant difference was detected for OS (Figure 3B).

**Figure 4.**
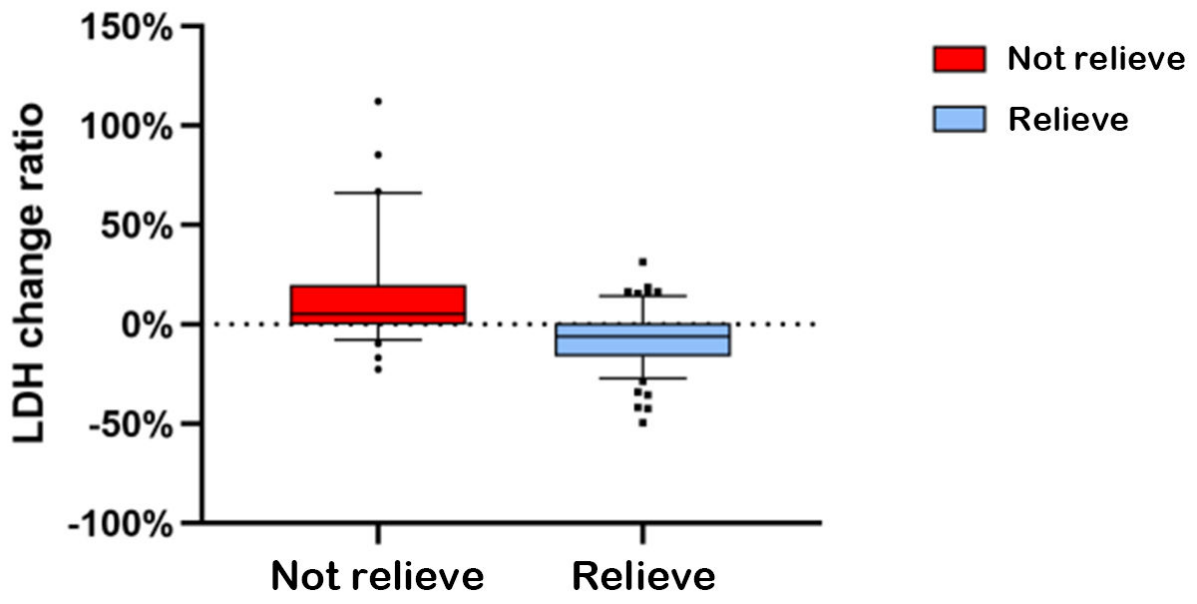
Comparison of LDH change rates between the tumor reduction group and the non-reduction group after 3 cycles of treatment. The tumor reduction group exhibited a significantly greater decrease in LDH levels compared with the non-reduction group (*P* < 0.05). (*P*<.001). “Relieve” indicates radiological evidence of reduction in the size of the primary tumor or affected lymph nodes after treatment, as assessed by the radiology department. “Not relieve” indicates no measurable reduction on follow-up imaging.

Further we analyzed the relationship between LDH dynamics and radiological tumor response. As shown in Figure 4, patients in the tumor reduction group experienced a significantly greater decline in LDH levels than those in the non-reduction group (*P*<.001; Figure 4). This indicates that a decrease in LDH levels is consistent with tumor shrinkage.

### Multivariate and Univariate Analyses

In the univariate analysis performed using the Cox proportional hazards model (Table 2), it was observed that patients with decreased LDH levels (threshold=200 U/L) following treatment demonstrated a 41% reduction in the risk of death compared with those exhibiting increased LDH levels (HR 0.59, 95% CI 0.37‐0.98; *P*=.04).

In addition to LDH changes, given that other clinical factors, including age, tumor length, tumor stage, tumor location, and the presence of comorbidities, were associated with PFS, we included these factors in our multivariate analysis (Table 2). The results of the multivariate analysis identified a reduction in LDH levels as an independent prognostic factor for improved outcomes (HR 0.59, 95% CI 0.36‐0.96; *P*=.04; Table 2).

Compared with patients with elevated LDH levels, LDH downregulation was an independent prognostic factor for a 41% reduction in the risk of death (HR 0.59, 95% CI 0.36‐0.96; *P*=.04).

**Table 2.** Univariate and multivariate analyses of factors influencing progression-free survival in all patients.

Variables	Univariate analysis			Multivariate analysis		
	HR^a^ (95% CI)		*P* value	HR (95% CI)		*P* value
Gender (male vs female)	0.73 (0.37‐1.43)		.35	—^b^		—
Age (years; <63 vs >63)	1.10 (0.67‐1.80)		.70	—		—
Comorbidity (yes vs no)	1.08 (0.65‐1.82)		.76	—		—
T stage (1‐3 vs 4)	0.6 (0.37‐0.97)		.04	0.67 (0.38‐1.20)		.18
N stage (0‐1 vs 2‐3)	0.78 (0.33‐1.80)		.56	—		—
Clinical stage (IVA^c^ vs IVB^d^)	0.60 (0.34‐1.08)		.09	0.75 (0.37‐1.48)		.40
Tumor length (<5 cm vs >5 cm)	0.98 (0.58-1.65)		.93	—		—
Tumor location						
Upper	Reference		—	—		—
Middle	0.69 (0.29‐1.65)		.40	—		—
Lower	0.74 (0.30‐1.82)		.51	—		—
LDH^e^ change (down vs up)	0.59 (0.37‐0.98)		.04	0.59 (0.36‐0.96)		.04

a HR: hazard ratio.

b Not available.

c IVA: cancer stage IVA.

d IVB: cancer stage IVB.

e LDH: lactate dehydrogenase.

## Discussion

### Principal Findings

This study provides valuable insights into the prognostic significance of dynamic changes in LDH levels among patients with ESCC undergoing ICI therapy. The findings demonstrate that a reduction in LDH levels is associated with improved PFS. Specifically, patients with a moderate LDH decrease (0% to 14.4%) exhibited a median PFS of 21.7 months, compared to 10.8 months in those with increased LDH. Multivariate analysis further confirmed that an LDH decrease independently predicted a 41% reduction in mortality risk. These results underscore the potential utility of dynamic LDH monitoring as an accessible, cost-effective, and valuable prognostic tool for informing individualized treatment strategies in advanced ESCC.

### Comparison to Previous Work

LDH, a key enzyme involved in cellular metabolism, facilitates the interconversion of lactate and pyruvate, which are critical to both aerobic and anaerobic metabolic pathways [13]. Elevated LDH levels have long been recognized as a prognostic marker in various malignancies, reflecting increased tumor burden and metabolic stress [14,15]. However, this study expands on existing knowledge by focusing on the dynamic changes in LDH levels during treatment rather than static measurements. This novel approach enables a more precise evaluation of treatment efficacy by capturing real-time fluctuations in tumor metabolism and therapeutic response. While traditional biomarkers for ICI therapy, such as PD-L1 expression, tumor mutational burden, and immune cell infiltration, provide valuable prognostic information, they often require complex, resource-intensive assessments [16-18]. In contrast, dynamic LDH monitoring offers a similarly predictive yet simpler and more cost-effective alternative. This is particularly advantageous in resource-limited settings, where such a practical tool can guide clinical decisions and optimize treatment strategies.

### Role of LDH in Tumor Metabolism and Therapeutic Implications

Elevated LDH activity in cancer cells reflects reliance on anaerobic glycolysis, leading to increased lactate production [19]. Accumulation of lactate acidifies the tumor microenvironment, disrupts immune cell function, promotes tumor progression, and creates conditions favorable for immune evasion [20,21]. Dynamic monitoring of LDH levels provides crucial insights into these metabolic shifts, illustrating the impact of therapy on tumor biology. Specifically, elevated lactate levels impair T-cell function, reduce immune cell cytotoxicity, and promote an immunosuppressive phenotype in tumor-associated macrophages [22,23]. Conversely, a reduction in LDH levels, indicative of decreased lactate production, may alleviate these immunosuppressive effects and enhance the efficacy of immune therapies. ICIs are specifically designed to counteract immune suppression in the tumor microenvironment [24]. Monitoring dynamic changes in LDH levels can therefore provide valuable insights into the evolving tumor state and its microenvironment during therapy. A decrease in LDH levels may signal effective tumor cell apoptosis or reduced glycolytic activity, both of which can relieve metabolic stress, enhance immune cell function, and ultimately improve therapeutic outcomes [25].

### Clinical Utility

Dynamic monitoring of LDH facilitates personalized treatment strategies by allowing real-time assessment of therapeutic efficacy and metabolic changes. Patients with significant reductions in LDH levels are more likely to benefit from continued ICI therapy, while those with stable or increasing LDH levels may require alternative therapeutic approaches [26]. This approach allows clinicians to optimize treatment strategies tailored to individual patient responses, enhancing treatment efficacy and patient outcomes. However, integrating LDH monitoring into routine clinical practice necessitates the establishment of standardized testing protocols and data interpretation guidelines to ensure consistency and reliability across clinical settings [27]. Regular LDH assessments should be incorporated into treatment plans to provide ongoing feedback on therapeutic response. Furthermore, combining LDH data with other biomarkers could enhance the predictive accuracy of treatment outcomes, supporting individualized decision-making and more precise treatment strategies [28].

### Limitations

Despite its potential, the retrospective design of this study introduces certain limitations, including selection bias and inconsistencies in data recording, which may impact the generalizability of the findings. Although efforts were made to minimize these biases through standardized procedures and data validation, the inherent limitations of retrospective analyses remain. In addition, the relatively small sample size may have influenced the statistical power and generalizability of the results. Moreover, variations in chemotherapy regimens could influence treatment outcomes, thereby affecting the conclusions of this study. In the future, additional samples should be collected to conduct more detailed subgroup analyses of various highly specific chemotherapy regimens, and prospective studies are needed to validate these findings and confirm the reliability of dynamic LDH monitoring as a prognostic marker. The applicability of these results to diverse populations and clinical settings also requires further exploration. Large-scale, multicenter studies are essential to evaluate the consistency and generalizability of LDH dynamics as a prognostic tool across diverse patient cohorts. Furthermore, variations across ethnicities, genders, and regions should be investigated to ensure broader applicability and relevance.

### Future Directions

Future research should include prospective cohort studies and randomized controlled trials to validate the prognostic value of dynamic LDH changes and identify optimal monitoring strategies for enhancing treatment efficacy. Exploring the combination of LDH with other biomarkers, such as genomic features and immune cell subsets, could further refine prognostic accuracy. Integrating multiple biomarkers may offer a more comprehensive assessment of patient status, informing more precise treatment strategies. Moreover, studies should investigate how to tailor immune therapies based on dynamic LDH changes, including potential treatment modifications and individualized approaches. Evaluating the impact of these strategies on long-term patient outcomes through clinical trials will be critical. The integration of LDH monitoring with other clinical data could lead to the development of comprehensive treatment plans, ultimately improving patient prognosis and quality of life.

### Conclusions

Dynamic changes in LDH levels, specifically a reduction within the 0% to 14.4% range, represent novel and significant predictors of PFS in patients with advanced ESCC. After 2 cycles of immunotherapy, patients with decreased LDH dynamic ratios demonstrated significantly better prognoses. These findings highlight the potential of LDH as a metabolic biomarker that reflects tumor biology and therapeutic response in real time. Future investigations are warranted to validate the utility of this specific LDH dynamic threshold as a prognostic tool. Such efforts could enhance personalized treatment strategies, ultimately improving clinical outcomes in patients with advanced ESCC.

## Abbreviations

ESCC: esophageal squamous cell carcinoma
HR: hazard ratio
ICI: immune checkpoint inhibitor
LDH: lactate dehydrogenase
OS: overall survival
PD-1: programmed death-1
PD-L1: programmed death-ligand 1
PFS: progression-free survival
